# Study on Ultrasonic Far-Infrared Radiation Drying and Quality Characteristics of Wolfberry (*Lycium barbarum* L.) under Different Pretreatments

**DOI:** 10.3390/molecules28041732

**Published:** 2023-02-11

**Authors:** Qian Zhang, Fangxin Wan, Yuanman Yue, Zepeng Zang, Yanrui Xu, Chunhui Jiang, Jianwei Shang, Tongxun Wang, Xiaopeng Huang

**Affiliations:** College of Mechanical and Electronical Engineering, Gansu Agricultural University, Lanzhou 730070, China

**Keywords:** ultrasound far-infrared, different pretreatment, wolfberry, drying kinetics, quality

## Abstract

In order to explore the effects of different pretreatment methods on the ultrasonic far-infrared synergistic drying characteristics and quality of wolfberry, the bioactive components (polysaccharide, total phenol, total flavonoids, and antioxidants), the quality characteristics (rehydration ratio, color, vitamin C content, and betaine content), and the microstructure of the dried products were used as evaluation indices to test wolfberry treated by five different pretreatments (hot blanching; candied pretreatment; NaOH solution treatment; NaCl solution treatment; and Na_2_CO_3_ solution treatment). The results showed that hot blanching pretreatment improved the drying rate and shortened the drying time, and that the vitamin C content of dried products pretreated by hot blanching (92.56 mg/100 g) was higher than that of dried products pretreated by other methods. All five pretreatment methods increased the contents of the total phenols, vitamin C, and betaine of wolfberry. Wolfberry treated by candied pretreatment had lower color differences and higher contents of polysaccharide (0.83 g/g), total phenol (9.26 mg/g), and total flavonoids (2.61 mg/g) than wolfberry treated by the other pretreatment methods. Wolfberry pretreated by NaCl solution had the strongest antioxidant capacity (65.01%). Wolfberry pretreated by Na_2_CO_3_ solution had the highest betaine content (3.24%). The observation of the microstructure of the dried products revealed that hot blanching caused the most damage to wolfberry, while the candied pretreatment was less destructive to the tissue cells of wolfberry. On the whole, the dried wolfberry products obtained by the candied pretreatment were of a better quality than products obtained by the other pretreatment methods.

## 1. Introduction

As a deciduous shrub of the Solanaceae family, wolfberry (*Lycium barbarum* L.) is widely distributed in China and other parts of Asia [1]. Wolfberry is rich in micronutrients and bioactive substances, including organic acids, phenols, carotenoids, and polysaccharides, and has antioxidant, anticancer, hypoglycemic, and lipid-lowering functions [2]. However, fresh wolfberry rots easily and mature wolfberry deteriorates after picking, so it is difficult to store and people rarely eat it directly after picking. Drying reduces the water activity and avoids microbial deterioration and a series of chemical reactions, thus extending the shelf life of wolfberry and reducing its weight for storage and transporting at relatively low costs.

Far-infrared drying has the advantages of a fast drying speed, low energy consumption, uniform heating, and little resulting damage to the materials. Although single far-infrared radiation has a significant positive effect on heat transfer in the drying process, it has little effect on mass transfer. Since ultrasonic strengthening promotes the mass transfer of materials, the combination of ultrasonic radiation and far-infrared radiation can simultaneously improve the heat and mass transfer of materials, resulting in a higher dehydration rate and better product quality. In recent years, direct-touch ultrasonic far-infrared drying technology has been successfully applied to jackfruit [3], banana [4], pumpkin [5], kiwifruit [6], and other agricultural products. 

The outer epidermis of wolfberry consists of a cuticle covered with a waxy layer, which severely hinders the evaporation of internal moisture during drying [7]; therefore, in the drying process for wolfberry, pretreatment by chemical or physical methods to dissolve the waxy layer should be considered to facilitate the removal of moisture and reduce the drying time. A previous study found that ultrasonic radiation with alkaline ethyl oleate pretreatment could significantly enhance the heat-pump drying rate for wolfberry and increase the rehydration of dried products [8]. The adoption of Na_2_CO_3_ pretreatment of wolfberry effectively shortened the drying time, accelerated the drying rate, improved the product’s color and rehydration rate, and increased the retention rate of the product’s nutrients [9]. Pretreatment with glycerol, maltodextrin, ascorbic acid, and sodium chloride concentrate, as an osmotic solution, obviously shortened the hot-air drying time, improved the texture characteristics, and facilitated the antioxidant capacity and total phenol content of the product [10].

These results showed that the drying efficiency and drying quality of *Lycium barbarum* were significantly improved by pretreatment. However, there have been few studies exploring the changes in quality resulting from the application of different pretreatment methods prior to the ultrasonic-assisted far-infrared drying of *Lycium barbarum*. In this study, the effects of blanching, sugaring pretreatment, NaOH solution treatment, NaCl solution treatment, and Na_2_CO_3_ solution treatment on the quality of ultrasonic-assisted far-infrared drying of *Lycium barbarum* were studied, with a focus on the drying kinetics, bioactive components (polysaccharides, total phenols, total flavonoids, and antioxidants), qualitative characteristics (color index, vitamin C, and betaine), and the microstructure of the dried product to provide a theoretical basis for improving the drying quality of wolfberry.

## 2. Results and Analysis

### 2.1. Analysis of Drying Characteristics

**Figure 1:**
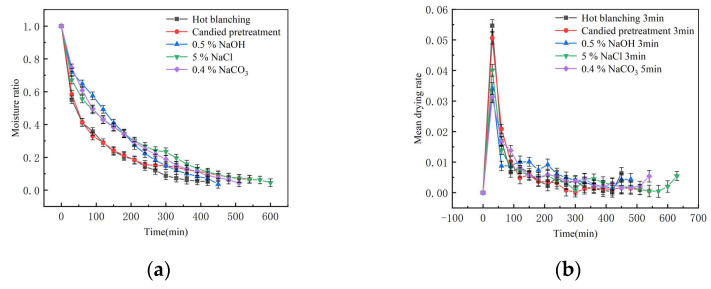
Drying curve (**a**) and drying rate curve (**b**) of wolfberry with different pretreatments.

Figure 1a,b show the effects of different pretreatment methods on the moisture ratio and drying rate of the direct ultrasonic vacuum far-infrared drying of wolfberries. The wax layer of wolfberry fruit is mainly composed of an organic mixture. Different pretreatments destroy the wax layer according to different principles, which causes different pretreatment effects. Specifically, the drying times required after different pretreatments such as hot blanching, candied pretreatment, NaOH solution treatment, and NaCl solution treatment were 420 min, 540 min, 450 min, and 600 min, respectively. Compared to the time required after 0.4% Na_2_CO_3_ treatment (510 min), the time required for hot blanching and NaOH solution treatment was reduced by 17.65% and 11.76%, respectively, while the time required for candied pretreatment and NaCl solution treatment increased by 5.9% and 17.65%, respectively. It can be seen that hot blanching damages the waxy layer on the surface of the wolfberry most seriously, followed by NaOH solution. This may be due to the relaxation and collapse of the plant cell structure induced by hot blanching and the destruction of cell walls and intercellular glial macromolecules in varying degrees during this process, increasing the permeability of the cell walls. In addition, the mechanical effect and cavitation effect caused by ultrasound reduce the adhesion of water, increases the ability of mass and heat transfer, and thus shorten the drying time [11]. After soaking in a strong alkali solution, the vegetable oil in wolfberries can be converted into alkali metal fatty acid salt, which will fill the wax layer gap in wolfberry epidermal cells. The hydrophilic groups in alkali metal fatty acid salt are arranged toward the gap. With the transpiration of epidermal moisture, the surface layer of the wolfberry seems to have multiple small channels similar to the cylinder, which destroys the wax layer of the wolfberry epidermis and makes the moisture of fresh wolfberries continuously discharged during ultrasonic far-infrared drying, thus improving the drying rate. The Na_2_CO_3_ solution treatment only makes the surface of the wolfberry wax layer thinner, so the drying effect is not as obvious as after the NaOH solution treatment. After the candied pretreatment solution, the hygroscopicity of sugar injected into wolfberry increases, and the concentration of sugar molecules in the material increases, which reduces the diffusion rate of water and increases the internal resistance to water movement during drying [12]. In addition, high extracellular solute concentrations do not lead to cell breakdown, which can result in massive dehydration and an accompanying contraction. This contraction may lead to a decrease in the transport properties of the cell structure, thereby increasing the time required to dry below the effective moisture level [13]. After NaCl solution treatment, the wolfberry itself continued to contact the salt solution. Due to the initial net flow of solute into the solution, the molecular size of NaCl was smaller than that of sucrose, and sugar was soaked into the structure of wolfberry, which increased the solid content in the sample, changed the permeability of the cell membrane, and thus limited the flow of water molecules [14].

### 2.2. Color Difference

The effects of different pretreatment drying on the color differences of wolfberry are shown in Table 1. The L*, a*, and b* of fresh wolfberries were 38.57, 39.39, and 26.02, respectively, as determined by a colorimeter. It was found that different pretreatment drying had little effect on the brightness value of the sample, and the L^*^ of the natural dried wolfberry was the closest to that of the fresh sample (38.57). Except for the treatment of Na_2_CO_3_ solution soaking, the a^*^ of the other pretreatments was higher than that of the fresh samples, indicating that these pretreatment methods improved the redness value of wolfberries and played a positive role in the color protection of wolfberry. This may be because the pretreatment before drying inhibited the enzymatic browning and non-enzymatic browning reactions during the sample drying process. By comparing the b^*^ values of different drying methods, it was found that the b* values were lower than those of the fresh samples, which significantly reduced the yellowness. The smaller the value of ΔE, the smaller the color difference. Comparing the different color difference values under different pretreatment methods, it was found that the dried finished product had the least color difference for the candied pretreatment samples. The color difference between natural drying and the Na_2_CO_3_ solution treatment is relatively large. This may be because the sample absorbed a large amount of solute in the process of sucrose impregnation, resulting in the transfer of low oxygen to the surface and reducing the discoloration of sugar-stained samples due to enzymatic browning [15]. Strong sunlight will degrade carotene, anthocyanin, and other pigments in wolfberries during the natural drying process; Na_2_CO_3_ solution treatment may activate the activity of related enzymes, resulting in various pigment degradation pathways. 

### 2.3. Rewaterability

As can be seen from Table 2, the best rehydration of the dried product obtained by natural drying was 2.76 g/g, which is due to the least damage to the cellular tissue structure by this process. The rehydration of dried wolfberries was 1.97 g/g, 1.53 g/g, 1.95 g/g, 1.91 g/g, and 1.64 g/g after hot blanching, candied pretreatment, 0.5% NaOH solution treatment, 5% NaCl solution treatment, and 0.4% Na_2_CO_3_ treatment, respectively. Compared with natural drying, the rehydration of dried wolfberries decreased by 31.36%, 44.57%, 32.06%, 33.45%, and 42.86%, respectively. Under all the different pretreatment methods, wolfberries dried by candied pretreatment had the worst rehydration capacity. This may be because a large number of sugar molecules were infiltrated into the wolfberry during the sugaring process, which reduced the water absorption rate of dry products. The rehydration ability is best after hot blanching treatment, which may be because wolfberry, after high-temperature bleaching, the most serious destruction of cell structure, resulted in the resistance of the water molecules in and out of the cell being greatly reduced, so dehydration is faster in the drying process, and water molecules into the cell rate are also fast in the rehydration process. 

### 2.4. Polysaccharides

The change in the polysaccharide content under different pretreatment conditions was analyzed (Table 2). The highest polysaccharide content of 0.83 g/g was found in the samples after the candied pretreatment, with an increase of 31.74% relative to natural drying (0.63 g/g). The polysaccharide content of the samples treated with hot blanching and NaOH solution was relatively low, at 0.54 g/g and 0.53 g/g, respectively, which was 14.29% and 15.87% lower than that of natural drying. This may be due to sugar molecules sugaring into the internal structure of wolfberries during the candied pretreatment; in addition, a large number of sucrose molecules were attached to the surface of wolfberries after the candied pretreatment. In the drying process, due to the cavitation effect and mechanical effect of ultrasonic, the microporous channel on the surface of wolfberry was increased, the surface sucrose molecules were promoted to enter the interior of wolfberry, and the content of polysaccharide in the dried samples was increased. The aggregation and sheer force of polysaccharides were destroyed during the blanching process, and the polysaccharides were decomposed into other small molecular substances. Furthermore, the addition of ultrasound increased the speed of the heat and mass transfer in wolfberries, and blanching severely damaged the wax layer on the berry’s surface, causing the internal temperature of the berry to rise rapidly, promoting the Maillard or caramel reaction of materials, and some polysaccharides would be converted into oligosaccharides and caramel, reducing the polysaccharide content. By analyzing the sugar content of the samples treated by NaCl solution and Na_2_CO_3_ solution, it was found that the content was 0.64 g/g and 0.74 g/g, respectively, which increased by 0.01% and 17.46% compared with the natural drying, indicating that the NaCl solution treatment had little effect on the content of polysaccharide, and Na_2_CO_3_ solution treatment increased the content of polysaccharide to a certain extent.

### 2.5. Total Phenols

The influence of different pretreatments on the total phenol content was analyzed (Table 2). The contents were all higher than those obtained by natural drying (4.45 mg/g), indicating that these five pretreatments had a positive effect on the retention of the total phenol content in wolfberry samples after direct ultrasonic far-infrared drying. The total phenol content of the samples treated with NaOH solution was 4.67 mg/g, which was a small increase of 4.9% relative to natural drying. This may be because when the sample was immersed in a strong alkaline solution, the alkaline solution neutralized the phenolic acid in the sample, resulting in a decrease in the total phenolic content. However, the strong alkaline solution seriously damaged the wax layer on the surface of wolfberries. The heat energy radiated by far-infrared was more easily absorbed by the moisture inside the material. The increase in temperature would lead to an increase in the contents of gallic acid and ferulic acid, thereby increasing the total phenolic acid content inside wolfberries. In comparison, the dried samples of wolfberry after being candied had the highest total phenolic content of 9.26 mg/g, which was more than twice as much as natural drying. This may lead to the increase in the soluble sugar content due to the entry of sucrose molecules in the sugaring process, and soluble sugar can induce the production of more polyphenols and ultimately maintain the balance of the flavanol and polyphenolic acid content [16]. The total phenol content after the blanching treatment was also high, which was 8.07 mg/g. This may be due to the fact that after blanching at high temperatures and drying at a high temperature, a large amount of salvianolic acid B was produced in wolfberries under the action of ultrasonic. With the drying process, salvianolic acid A would be degraded, and salvianolic acid A would also undergo a cyclization reaction to generate iso-salvianolic acid C and salvianolic acid C, and finally more phenolic compounds would accumulate [17].

### 2.6. Total Flavonoids

The analysis of the total flavonoid content data in Table 2 showed that the highest flavonoid content of 2.61 mg/g was found in samples after they were candied, which was 55.36% higher than that obtained by natural drying (1.68 mg/g). This may be because the sugar stain in the sucrose solution, which has a large viscosity, would form a resistance on the surface of wolfberries, hindering the dissolution of the total flavonoids. Furthermore, the infrared wave emitted by the infrared emission tube penetrated the cells during the drying process, breaking the covalent bond between the polymers in wolfberries, releasing a large number of flavonoids, and increasing the retention rate of the flavonoids content. After blanching, NaOH solution treatment, NaCl solution treatment, and Na_2_CO_3_ solution treatment, the total flavonoids content of the samples was lower than that obtained by natural drying, which was reduced by 35.71%, 18.45%, 26.19%, and 15.48%, respectively. This may be because different pretreatments caused different degrees of damage to the structure and activity of related flavonoids synthases, resulting in the inability to synthesize some flavonoids. Therefore, blanching caused the greatest amount of damage to them. During the whole drying process, the samples were in contact with oxygen, and the drying with air as the drying medium would make some flavonoids oxidized and degraded, thereby reducing the content of the total flavonoids.

### 2.7. Antioxidant Activity

Analysis of the antioxidant capacity of wolfberry after drying with different pretreatments revealed (Table 2) that the pretreatment with the worst antioxidant capacity (25.58%) was hot blanching, which decreased by 32.80% compared to natural drying (33.97%). Probably because some water-soluble phenolic acid compounds are unstable in nature and are susceptible to decomposition, oxidation, and dehydroxylation by heat during high temperature rinsing, and the increase in the antioxidant capacity substances due to the Maillard reaction caused by the drying process does not compensate for the destruction of the phenolic substances [18]. The antioxidant capacity of LBP dried after pretreatment in other ways was higher than that of natural drying, probably because a heat treatment after different pretreatments may destroy the cell wall, causing the release of phenolic compounds from the insoluble part of the sample, and, in addition, far-infrared has the ability to break down covalent bonds, releasing a variety of active substances such as flavonoids, carotenoids, tannins, ascorbic acid, flavoproteins, or polyphenols from the repetitive polymers, which by the direct quench or inhibition of free radicals, provision of protons or electrons, etc., together exert antioxidant effects.

### 2.8. Vitamin C

Analysis of the Vc content obtained after drying the samples with different pretreatments (Table 2) revealed that the vitamin C content obtained after treatment with Na_2_CO_3_ solution (57.22 mg/100 g) did not change significantly compared with that obtained by natural drying (58.15 mg/100 g), indicating that the treatment had a small degree of effect on the Vc content. In comparison with the other treatments, the content was higher than that obtained by natural drying, with 92.56 mg/100 g, 83.72 mg/100 g, 71.96 mg/100 g, and 61.73 mg/100 g, respectively, an increase of 59.17%, 43.97%, 23.75%, and 6.15%, respectively. Among them, the blanching treatment and NaOH solution treatment have a positive effect on the vitamin C in the sample. It may be because the drying rate of the sample after pretreatment by these two methods is fast, and the time required for drying to below the safe moisture content is short. The oxidation degree of ascorbic acid is small, and the Vc content is retained to a certain extent. The treatment with candied and NaCl solutions increased the retention of their contents because during the candied process, immersion in sucrose solution increased the viscosity of the surface of wolfberry, effectively hindering the spillage of Vc. After the salt solution immersion, NaCl molecules penetrated inside the sample, increasing the content of solids and changing the permeability of the cell membrane, similarly inhibiting the spillage of Vc.

### 2.9. Betaine

It can be seen from Table 2 that the five different pretreatment methods have a positive effect on the retention of betaine in wolfberry, and the content of betaine in the samples treated by blanching, candied pretreatment, and NaOH solution is 2.87%, 2.84%, and 2.87%, respectively. The influence degree is very small, which is increased by 9.5%, 8.5%, and 9.5% compared with the natural drying. In comparison to other pretreatment methods, dried wolfberries treated with Na_2_CO_3_ solution have the highest betaine content, at 3.24%. This may be because betaine is a kind of amphoteric ion similar to amino acid, which is very unstable and easy to decompose. A weak alkali solution can be stabilized by soaking, and the addition of ultrasonics improves the mass and heat transfer coefficients during the drying process. The radiation absorbed by wolfberries is relatively higher, the drying time is accelerated, and more betaine components are retained.

### 2.10. Microstructure

**Figure 2:**
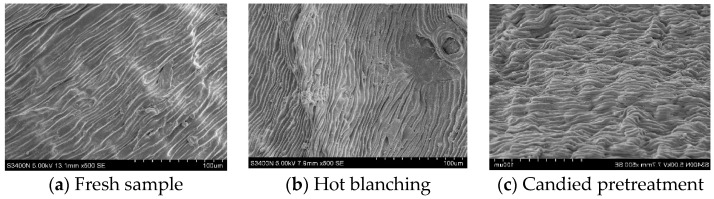

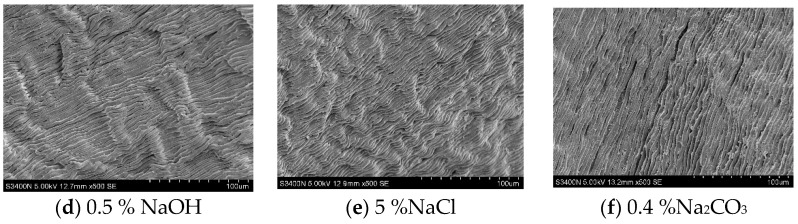
Effect of pretreatment methods on microstructure of wolfberry by ultrasonic far-infrared synergistic drying.

The effects of drying with different pretreatment methods on the microstructure of the samples are shown in Figure 2. The wax layer of wolfberries was loosely arranged in a flocculent shape, and the arrangement between the strips was regular. The surface was rough, and there was an obvious depression. In contrast, the surface texture of fresh wolfberries was relatively regular. After different pretreatments, the wax layer was damaged to different degrees. The comparison revealed that the damage of blanching on the surface of wolfberries was the most obvious. After high temperature blanching, the warp and weft structures of ‘cellulose microfibre-hemicellulose-pectin’ in the cell wall of wolfberries will be destroyed. In severe cases, the epidermis will be scalded, and more micropore channels will be formed with ultrasound, which increases the heat and mass transfer rates of the sample and accelerates the water transfer. It was found from Figure 2c,e that more fine lines were generated on the surface of the sample after treatment with sugar and NaCl solutions, accompanied by many irregular protrusions and confusion. This may be due to the residual solid on the surface during the impregnation of sugar and salt solutions. It was difficult to discharge water during the drying process. Under the vibration of an ultrasonic vibrator, water molecules were irregularly arranged on the surface. The observation Figure 2d found that the surface of wolfberries soaked in a strong alkali solution also showed different degrees of protrusions, compared with sugared and salted protrusions, which were significantly reduced. It can be seen from Figure 2f that the Na_2_CO_3_ solution treatment was inferior to other pretreatments. The epidermal structure had small gaps, a compact structure, and increased surface wrinkles, which indicated that the pretreatment method was helpful in forming a uniform organizational structure on the surface.

## 3. Materials and Methods

### 3.1. Test Materials

The wolfberries were obtained in the first ten days of August from the variety Ningqi No. 1 in the Ningxia Zhongning Wolfberry Plantation (Ningxia, China). The wolfberries used in the experiment were not treated in any way and were placed in a refrigerator at 4 °C for later use. The average moisture content of the fresh wolfberry, determined by the AOAC official method at 105 °C for 24 h, was 79.5% [19].

### 3.2. Test Equipment

The direct-touch ultrasonic far-infrared drying equipment used in this experiment was jointly developed by the School of Mechanical and Electrical Engineering of Gansu Agricultural University (Lan Zhou, China) and Tianshui Shenghua Microwave Technology Co., Ltd. (Tian Shui, China). The specific structure and parameters are described in the literature [20].

### 3.3. Test Method

#### 3.3.1. Pretreatment Conditions

A previous experimental study showed that ultrasonic-assisted far-infrared drying technology played a significant role in strengthening the heat- and mass-transfer processes of wolfberry drying [12]. In this experiment, five different pretreatment methods were adopted to study the drying of wolfberry. The five methods are as follows: hot blanching (soaking in boiling water for 3 min); candied pretreatment (soaking in a 30% sucrose solution for 30 min); NaOH solution treatment (soaking in a 0.5% NaOH solution for 3 min); NaCl solution treatment (soaking in a 5% NaCl solution for 3 min); and Na_2_CO_3_ solution treatment (soaking in a 0.4% Na_2_CO_3_ solution for 5 min).

#### 3.3.2. Test Process

**Figure 3:**
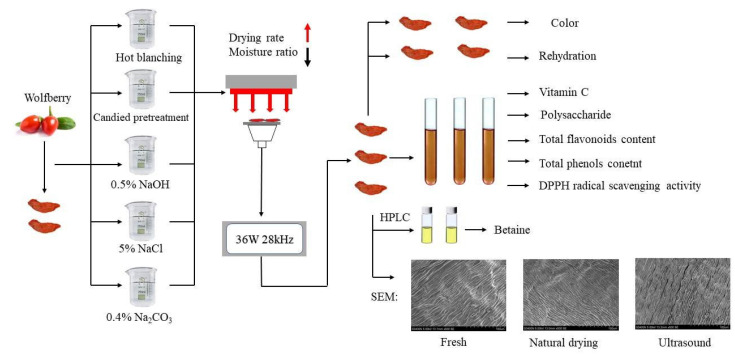
Schematic diagram of direct ultrasonic far-infrared drying and quality detection of wolfberry under different pretreatment methods.

Samples of the same size and moisture content were chosen as the test materials. On the basis of preliminary experiments, the ultrasonic power of 36 W, ultrasonic frequency of 28 kHz, irradiation height of 250 mm, infrared power of 900 W, and drying temperature of 50 °C were selected. One hour before the experiment, the samples were removed from the low-temperature refrigerator and washed with water once the surface temperature of the samples reached room temperature (22 ± 1 °C). The samples were washed with water. Each pretreatment experiment weighed 100 ± 0.5 g. Before drying, the equipment was set in advance at a specific temperature to preheat for 30 min, then the weighed materials were laid flat on the vibrating plate, and finally the door of the drying box was closed. The drying process was weighed every 20 min, and the moisture content of the wolfberry at the sampling time point was calculated according to the mass loss until it dropped below the safe moisture content (10%) to stop the test. All the tests involved in this experiment are shown in Figure 3.

### 3.4. Measurement of Test Indicators

#### 3.4.1. Determination of Moisture Content

(1) $$X=\frac{M_{t}-M_{d}}{M_{d}}$$

where X represents the dry basis moisture content, %; *M_t_* represents the weight of the wolfberry at time *t*, g; and *M_d_* represents the dry weight of the wolfberry, g.

#### 3.4.2. Determination of Drying Rate

(2) $$\mathrm{DR}=\frac{M_{t_{2}}-M_{t_{1}}}{t_{2}-t_{1}}$$

where DR represents the drying rate, g/s; $M_{t_{2}}$ and $M_{t_{1}}$ represent the weight of the wolfberry at *t*_2_ and *t*_1_, g; and *t*_2_ − *t*_1_ represents the time interval of two weighing, min.

#### 3.4.3. Determination of Moisture Ratio

(3) $$\mathrm{MR}=\frac{M_{t}-M_{e}}{M_{c}-M_{e}}$$

where *M_t_* represents the dry base moisture content of the wolfberry at t time, %; *M_c_* represents the initial moisture content of the wolfberry, %; and *M_e_* represents the moisture content of the dried wolfberry to equilibrium, %.

Due to the small equilibrium water content of wolfberry, the simplified Equation chosen here is:

(4) $$\mathrm{MR}=\frac{M_{t}}{M_{c}}$$

### 3.5. Determination of Drying Quality

#### 3.5.1. Determination of Rehydration Ratio

(5) $$\;R_{f}=\frac{G_{f}}{G_{g}}$$

where *R_f_* represents the water ratio; *G_f_* denotes the quality of the surface water removed by the wolfberry after rehydration; and *G_g_* represents the quality of the wolfberry before rehydration.

#### 3.5.2. Determination of Color

The surface color of the wolfberry was measured by a precision colorimeter (CS-210, Zhengzhou North–South Instruments and Equipment Co., Ltd., Zhengzhouu, China). The results were expressed as L*, a*, and b* values, and the total color difference (ΔE) was calculated according to Equation (6).

(6) $$\Delta E=\sqrt{{\left(L^{*}-L_{0}^{*}\right)}^{2}+{\left(a^{*}-a_{0}^{*}\right)}^{2}+{\left(b^{*}-b_{0}^{*}\right)}^{2}}$$

where ∆E represents the total color difference of the sample; L^*^, a^*^, and b^*^ represent the brightness, red-green, and yellow-blue values of fresh wolfberry, respectively; and $L_{0}^{*}$, $a_{0}^{*}$, and $b_{0}^{*}$ represent the brightness, red-green, and yellow-blue values of the dry products, respectively.

#### 3.5.3. Determination of Polysaccharide Content

The preparation of the extract changed slightly in the research methods of Wan et al. [21]. After precision weighing of 0.5 g of dried wolfberry products in an ice bath and grinding them into pulp, the material–liquid ratio was added to 25 mL of anhydrous ethanol at a ratio of 1:5 (m/V) in a 50 mL stoppered triangular flask and extracted by ultrasonication at 40 kHz, 100 W, and 65 °C for 40 min, followed by centrifugation at 4 °C and 4000 r/min for 10 min, taking its supernatant with an anhydrous ethanol constant volume to 25 mL standby. In the experiment, 5 μL of the extract was taken, and the polysaccharide content was determined by the phenol sulfate method [22]. The sucrose standard curve (y = 0.00823x − 0.159, r^2^ = 0.99355) was obtained. The polysaccharide content of the dried wolfberry products was calculated using Equation (7):

(7) $$\mathrm{Polysaccharide}\;\mathrm{content}=\frac{V_{2}C_{1}}{V_{1}M}$$

where *C*_1_ represents the content of sucrose in the sample determination tube obtained on the standard curve, μg; *V*_1_ represents the volume of the sample extract at titration, mL; *V*_2_ represents the total volume of the sample extract, mL; and *M* represents the sample mass, g.

#### 3.5.4. Determination of Total Phenol Content

The preparation of the extract was changed slightly based on the research methods of Wan et al. [21]. After the precision weighing of 0.5 g of wolfberry dried products in ice bath conditions and grinding them into pulp, the material–liquid ratio was added to 25 mL of anhydrous ethanol at 1:5 (m/V) in a 50 mL stoppered triangular flask and extracted by ultrasonication at 50 kHz, 100 W, and 60 °C for 35 min, followed by centrifugation at 4 °C and 4000 r/min for 10 min, taking its supernatant with an anhydrous ethanol constant volume to 25 mL standby. In the experiment, 300 μL of the extract was taken, and the total phenol content was determined by the Folin–Ciocalteu method [23]. The gallic acid standard curve (y = 0.0489x + 0.5048, r^2^ = 0.9905) was obtained. The total phenol content of dried wolfberry products was calculated using Equation (8):

(8) $$\mathrm{Total}\;\mathrm{phenol}\;\mathrm{content}=\frac{V_{2}C_{2}}{V_{1}M}$$

where *C*_2_ represents the content of gallic acid in the sample determination tube obtained from the standard curve, μg; *V*_1_ represents the volume of the sample extract used in the titration, mL; *V*_2_ represents the total volume of the sample extract, mL; and *M* represents the sample mass, g.

#### 3.5.5. Determination of Total Flavonoids Content

The preparation of the extract was changed slightly based on the research methods of Wan et al. [21]. After the precision weighing of 0.5 g of wolfberry dried products in ice bath conditions and grinding them into pulp, and the material–liquid ratio was added to 25 mL of anhydrous ethanol at 1:5 (m/V) in a 50 mL stoppered triangular flask and extracted by ultrasonication at 20 kHz, 100 W, and 60 °C for 35 min, followed by centrifugation at 4 °C and 4000 r/min for 10 min, taking its supernatant with an anhydrous ethanol constant volume to 25 mL standby. In the experiment, 1200 μL of the extract was taken, and the total flavonoids content was determined by the NaNO_2_-Al (NO_2_)_3_-NaOH method [24]. The catechin standard curve (y = 0.0049x + 0.04, r^2^ = 0.9984) was obtained. The content of the total flavonoids content in the dried wolfberry products was calculated according to Equation (9):

(9) $$\mathrm{Total}\;\mathrm{flavonoids}\;\mathrm{content}=\frac{V_{2}C_{3}}{V_{1}M}$$

where *C*_3_ represents the amount of catechin contained in the sample determination tube from the standard curve, μg; *V*_1_ represents the volume of the sample extract used in the titration, mL; *V*_2_ represents the total volume of the sample extract, mL; and *M* represents the sample mass, g.

#### 3.5.6. Determination of Antioxidant Activity

The preparation of the extract was changed slightly based on the research methods of Wan et al. [21]. A total of 1 g of the sample was weighed out and placed in the mortar, 50 mL ethanol was added according to the material–liquid ratio of 1:5 (m/V), it was grinded into a slurry under ice bath conditions, and transferred into a 50 mL centrifuge tube. Under light-proof conditions, the sample was shaken in a constant temperature shaker (120 r/min) for 48 h and then centrifuged at 4 °C and 4000 r/min for 10 min. The supernatant was taken and fixed with anhydrous ethanol to 25 mL for standby. After 80 μL was taken, the antioxidant capacity of the organic active substances was determined by the DPPH method [25]. Calculate the antioxidant capacity of dried wolfberry products according to (10):

(10) $$\mathrm{Antioxidant}\;\mathrm{capacity}=\frac{A_{0}-A}{A_{0}}\times 100\%\;$$

where *A* is the absorbance value of the sample solution and *A*_0_ is the absorbance value without the sample solution.

#### 3.5.7. Determination of Vc Content

Weigh 1.0 g of the sample in a mortar, add 20 mL of 50 g/L trichloroacetic acid solution (TCA solution), grind into a slurry in an ice bath, transfer to a 100 mL volumetric flask, fix the volume to the scale with 50 g/L TCA solution, mix, centrifuge for 10 min at 4 °C and 1800 r/min, and take the supernatant as a reserve. The content of Vc was determined by spectrophotometry after absorbing 1000 μL. The ascorbic acid standard curve (y = 35.39x − 1.5731, r^2^ = 0.9984) was obtained, and the results were expressed as the ascorbic acid equivalent (mg GAE)/100 g on the dried base of wolfberry. The content of ascorbic acid in the dried *Lycium barbarum* product was calculated according to (11):

(11) $$\mathrm{Vc}\;\mathrm{content}=\frac{V\times m^{\prime }}{V_{N}\times m\times 1000}\times 100(\mathrm{mg}/100\;g)\;$$

where $m^{\prime }$ represents the mass of ascorbic acid obtained from the standard curve, μg; V represents the volume of the sample extract used in titration, mL; V_N_ represents the total volume of the sample extract, mL; and m represents the sample quality, g.

#### 3.5.8. Determination of Betaine Content

(I). Chromatographic conditions

Chromatographic column: Merk RP-C 18 (250 mm × 4.6 mm, 5 μm); mobile phase: acetonitrile–water (83:17, V/V); flow rate: 1 mL/min; column temperature: 30 °C; detection wavelength: 195 nm; injection volume: 1 μL.

(II). Preparation of reference solution

The appropriate amount of betaine was weighed accurately, and a certain amount of methanol was added to prepare a reference reserve solution with a mass concentration of 1 mg/mL. A total of 0.5 mL of the above reference stock solution was accurately measured, diluted with 0.5 mL of methanol, and shaken well to obtain the reference solution with a mass concentration of 250 μg/mL.

(III). Preparation of test samples

A total of 1.0 g of the sample was accurately weighed and placed in a mortar. A total of 30 mL of methanol solution was added and grinded into slurry under ice bath conditions. The slurry was extracted according to a certain method, filtered and centrifuged in a 10 mL centrifuge tube. The supernatant was filtered through a 0.45 μm membrane and the subsequent filtrate was taken as the test solution.

### 3.6. Microstructure

The microstructure of dried wolfberries under different pretreatment conditions was observed by SEM. The dry sample was cut into 3 mm × 3 mm pieces, fixed on the sample table with conductive tape, coated for 50 s, reached an accelerated voltage of 5.0 kv, and was magnified 500 times for the acquisition image.

### 3.7. Statistical Analysis

All results were expressed as the mean ± standard deviation of three independent experiments. The curve and histogram were drawn by Origin 8.0. SPSS 24.0 was used for the analysis of variance (ANOVA). Tukey ‘s multiple-range test was used to analyze the significance of the mean difference, and the significance level was 0.05.

## 4. Conclusions

In this paper, the ultrasonic synergistic far-infrared drying characteristics and quality changes of wolfberry under different pretreatments were investigated. The results showed that the drying rate curves increased first and then decreased; the effects of hot blanching and the NaOH solution treatment were the most significant, and the time used was significantly shortened compared with other pretreatment methods. In general, the candied treatment effectively improved the quality of dried products with the lowest total color difference and the highest polysaccharide, total phenol, and total flavonoid contents of 0.83 g/g, 9.26 mg/g, and 2.61 mg/g, respectively. Compared with natural drying, the five pretreatment methods increased the contents of total phenols, vitamin C, and betaine in wolfberry, and the vitamin C content of dried products after the blanching treatment was the highest (92.56 mg/100 g); the betaine content of the samples treated by Na_2_CO_3_ solution was the highest (3.24%); and the antioxidant capacity of dried wolfberry treated by NaCl solution was the strongest (65.01%). By comparing the microstructure diagrams of the samples after drying with different pretreatments, it was found that different methods damaged the waxy layer of the epidermis of wolfberry to different degrees, and hot scalding damaged it most severely, reducing the resistance to moisture transfer and thus shortening the drying time. The damage to wolfberry tissue cells by sugar and salt treatment was small, but the increase in the intracellular solute hindered the diffusion of free water. To sum up, the candied pretreatment has better quality characteristics than other pretreatment methods. The results from this work will provide guidelines for selecting a drying method that maximizes the retention of product qualities and facilitates the industrial production of wolfberries.

It is worth emphasizing that in this paper, the drying characteristics and quality characteristics of different pretreatment ultrasounds combined with far-infrared drying wolfberry were studied, but the more specific heat and mass transfer process inside wolfberries remain unclear. In future research, the heat and mass transfer mechanisms of wolfberries should be further studied by simulation to provide a theoretical basis for the optimization of the later drying process.

## Figures and Tables

**Table 1:** Effect of different pretreatment drying on color difference of wolfberry samples.

Processing Mode	L*	a*	b*	ΔE
Fresh sample	38.57 ± 0.87 ^a^	39.39 ± 0.59 ^ab^	26.02 ± 0.27 ^a^	-
Natural drying	38.47 ± 2.21 ^a^	43.93 ± 2.67 ^a^	19.38 ± 0.74 ^by^	8.56 ± 1.41 ^b^
Hot blanching	38.11 ± 0.99 ^a^	41.99 ± 2.36 ^ab^	19.80 ± 0.35 ^b^	7.19 ± 0.79 ^bc^
Candied pretreatment	39.17 ± 0.09 ^a^	39.53 ± 2.34 ^ab^	20.00 ± 0.57 ^b^	6.47 ± 0.76 ^c^
0.5% NaOH	38.88 ± 1.31 ^a^	39.88 ± 2.62 ^ab^	19.51 ± 0.85 ^bc^	7.19 ± 0.48 ^bc^
5% NaCl	38.43 ± 1.30 ^a^	39.75 ± 2.45 ^ab^	19.11 ± 0.52 ^bc^	7.44 ± 0.79 ^bc^
0.4% Na_2_CO_3_	37.90 ± 0.07 ^a^	36.77 ± 0.65 ^b^	18.79 ± 0.61 ^b^	8.58 ± 0.43 ^a^

Note: Values followed by different letters in each column indicated significant differences (* *p* < 0.05).

**Table 2:** Effects of different pretreatment drying on the quality changes of wolfberry samples.

Processing Mode	Rewaterability g/g	Polysaccharide g/g	Total Phenols mg/g	Total Flavonoids mg/g	Antioxidant Activity %	Vc mg/100 g	Betaine %
Natural drying	2.76 ± 0.14 ^a^	0.63 ± 0.01 ^c^	4.45 ± 0.04 ^f^	1.68 ± 0.05 ^b^	33.97 ± 0.13 ^d^	58.15 ± 0.68 ^e^	2.62 ± 0.05 ^d^
Hot blanching	1.97 ± 0.01 ^b^	0.54 ± 0.02 ^d^	8.07 ± 0.04 ^b^	1.08 ± 0.03 ^e^	25.58 ± 0.69 ^e^	92.56 ± 0.39 ^a^	2.87 ± 0.11 ^c^
Candied pretreatment	1.53 ± 0.03 ^d^	0.83 ± 0.02 ^a^	9.26 ± 0.03 ^a^	2.61 ± 0.04 ^a^	58.81 ± 1.22 ^c^	83.72 ± 0.43 ^b^	2.84 ± 0.06 ^c^
0.5% NaOH	1.95 ± 0.01 ^b^	0.53 ± 0.01 ^d^	4.67 ± 0.05 ^e^	1.37 ± 0.06 ^c^	62.08 ± 0.06 ^b^	71.96 ± 0.89 ^c^	2.87 ± 0.05 ^c^
5% NaCl	1.91 ± 0.02 ^b^	0.64 ± 0.03 ^c^	5.68 ± 0.07 ^d^	1.24 ± 0.04 ^d^	65.01 ± 0.73 ^a^	61.73 ± 0.88 ^d^	3.08 ± 0.05 ^b^
0.4% Na_2_CO_3_	1.64 ± 0.02 ^c^	0.74 ± 0.02 ^b^	7.16 ± 0.06 ^c^	1.42 ± 0.03 ^c^	61.11 ± 0.81 ^b^	57.22 ± 0.72 ^e^	3.24 ± 0.07 ^a^

Note: Data are expressed as means ± standard deviation of triplicate samples. The letters in the same column reveal significant differences (*p* < 0.05) according to the Duncan test.

## Data Availability

The authors confirm that the data supporting the findings of this study are available within the article.
